# Transcriptome-Wide N6-Methyladenosine Alternations in Pulmonary Arteries of Monocrotaline-Induced Pulmonary Arterial Hypertension in Rats and Novel Therapeutic Targets †

**DOI:** 10.3390/biomedicines12020364

**Published:** 2024-02-04

**Authors:** Yilu Feng, Zaixin Yu, Mi Tang, Jiang Li, Baohua Peng, Mukamengjiang Juaiti, Yiyang Tang, Benhui Liang, Mingqi Ouyang, Qingqing Liu, Jie Song

**Affiliations:** 1 Department of Cardiology, Xiangya Hospital, Central South University, Changsha 410008, China; yilu786557320@163.com (Y.F.); yuzaixin@126.com (Z.Y.); 208112182@csu.edu.cn (B.P.); docmkmj@163.com (M.J.); tangyiyang@csu.edu.cn (Y.T.); xyliangbh@163.com (B.L.); 2 Research Institute of Blood Lipid and Atherosclerosis, Central South University, Changsha 410011, China; lijiangcs@163.com (J.L.); oymqxy@csu.edu.cn (M.O.); 3 Department of Cardiovascular Medicine, The Second Xiangya Hospital, Central South University, Changsha 410011, China; 4 Department of Cardiovascular Surgery, The Second Xiangya Hospital, Central South University, Changsha 410011, China; tangmi1129@163.com; 5 Department of Respiratory and Critical Care, The Second Xiangya Hospital, Central South University, Changsha 410011, China; lqq.aileen@csu.edu.cn

**Keywords:** N6-methyladenosine, pulmonary arterial hypertension, centromere protein F, leucine rich pentatricopeptide repeat containing, pulmonary artery smooth muscle cells

## Abstract

N6-methyladenosine (m^6^A) is a post-transcriptional epigenetic change with transcriptional stability and functionality regulated by specific m^6^A-modifying enzymes. However, the significance of genes modified by m^6^A and enzymes specific to m^6^A regulation in the context of pulmonary arterial hypertension (PAH) remains largely unexplored. MeRIP-seq and RNA-seq were applied to explore variances in m^6^A and RNA expression within the pulmonary artery tissues of control and monocrotaline-induced PAH rats. Functional enrichments were analyzed using the Gene Ontology and Kyoto Encyclopedia of Genes and Genomes. To screen candidate m^6^A-related genes, the STRING and Metascape databases were used to construct a protein–protein interaction network followed by a real-time PCR validation of their expression. The expression level of an m^6^A regulator was further investigated using immunohistochemical staining, immunofluorescence, and Western blot techniques. Additionally, proliferation assays were conducted on primary rat pulmonary artery smooth muscle cells (PASMCs). We identified forty-two differentially expressed genes that exhibited either hypermethylated or hypomethylated m^6^A. These genes are predominantly related to the extracellular matrix structure, MAPK, and PI3K/AKT pathways. A candidate gene, centromere protein F (*CENPF*), was detected with increased expression in the PAH group. Additionally, we first identified an m^6^A reader, leucine rich pentatricopeptide repeat containing (LRPPRC), which was downregulated in the PAH rat model. The in vitro downregulation of *Lrpprc* mediated by siRNA resulted in the enhanced proliferation and elevated expression of *Cenpf* mRNA in primary rat PASMCs. Our study revealed a modified transcriptome-wide m^6^A landscape and associated regulatory mechanisms in the pulmonary arteries of PAH rats, potentially offering a novel target for therapeutic strategies in the future.

## 1. Introduction

Pulmonary arterial hypertension (PAH) is an irreversible cardiopulmonary vascular disease caused by a progressive increase in pulmonary vascular resistance, eventually leading to high pulmonary artery pressure and right heart failure [1]. The main causes of high pressure of the pulmonary arteries include vasoconstriction, pulmonary vascular remodeling, and extracellular matrix (ECM) deposition [2]. Pulmonary vascular remodeling, mainly caused by the excessive proliferation and apoptosis resistance of pulmonary artery smooth muscle cells (PASMCs), is the basis for developing PAH [3]. Current treatments for PAH center on decreasing pulmonary vasoconstriction, rather than reversing vascular remodeling [4]. The disease inevitably worsens and results in a high mortality rate. Therefore, identifying a new therapeutic target to reverse vascular remodeling is extremely urgent.

PAH is a complex disease with multiple contributing factors, including genetic, environmental, and epigenetic elements [5]. Epigenetic changes in the pathogenesis of vascular remodeling have been previously demonstrated, but so far little is known about the specific epigenetic targets for reversing PAH [6]. Over the past decades, increasing attention has been paid to RNA modification with the rapid development of high-throughput sequencing. Up to now, over 170 distinct RNA modifications have been identified, including N1-methyladenosine (m^1^A), 7-methylguanosine (m^7^G), N6-methyladenosine (m^6^A), and 5-methylcytosine (m^5^C). Among these, m^6^A is the most abundant and prevalent internal chemical modification found in the mRNA of a wide range of eukaryotes [7,8]. Notably, m^6^A is pivotal in controlling RNA splicing, translation, localization, and stability [9]. It also participates in several biological functions, including cell proliferation, metabolism, regeneration, and differentiation [10]. Our previous study indicated that genetic modifiers called “second hits” may trigger the onset of PAH [11]. Therefore, epi-transcriptional changes can also serve as potential modifying factors in this manner contributing to the pathogenesis of PAH.

M^6^A is dynamic and reversible and regulated by methyltransferases (also called “writers”), demethylases (also called “erasers”), and different m^6^A-binding proteins (also called “readers”) [12]. The formation process of m^6^A is catalyzed by methyltransferases, while the removal process is mediated by demethylases. In addition, a group of specific RNA-binding proteins can recognize m^6^A motifs, thus influencing corresponding m^6^A functions [13,14]. Readers that have been reported so far are the YT521-B homology (YTH) domain, Heterogeneous Nuclear Ribonucleoprotein A2/B1 (HNRNPA2B1), and the insulin-like growth factor 2 mRNA-binding protein (IGF2BP) domain and leucine rich pentatricopeptide repeat containing (LRPPRC) [15,16]. Gaining a more profound insight into these regulators is crucial to uncovering the function and mechanism of m^6^A in post-transcriptional regulation [17]. Increasing evidence suggests that imbalances in the expression and genetic alterations of m^6^A regulators are associated with various biological process dysfunctions, including apoptosis/proliferation, developmental anomalies, aggressive tumor progression, reduced self-renewal ability and immune system irregularities [18,19]. Accumulating evidence suggests that the dysregulation of m^6^A enzymes is involved in the pathogenesis of PAH [20]. It has been reported that YTHDF2 levels are significantly increased in PASMCs [21]. Furthermore, YTHDF2 recognizes METTL3-mediated m^6^A-modified *PTEN* mRNA and decreases PTEN levels, which leads to the proliferation of PASMCs by activating the PI3K/AKT signaling pathway [21]. Nevertheless, the roles of m^6^A regulators in PAH have not been elucidated.

The influence of m^6^A and its regulatory enzymes differs among various tissues and cell types [10]. As mentioned earlier, the main pathological manifestation of PAH is vascular remodeling. However, only a few recent studies have reported the possible m^6^A in PAH rat lung tissues, but the change in the m^6^A profile in PAH pulmonary artery tissues remains unclear [22,23]. Therefore, understanding the tissue-specific role of m^6^A among vascular constituent cells is essential to fully explore its role in PAH. Moreover, given the progressive nature of PAH, conducting longitudinal studies to investigate m^6^A over time is crucial for grasping its dynamics in the development of PAH [24]. However, this study is complicated by heterogeneity in the causes and clinical presentation of PAH patients. Currently, linking basic research outcomes with clinical practice remains a widespread challenge across numerous scientific disciplines. Therefore, examining the translational possibilities of these findings for creating diagnostic methods or tailored treatments is of significant importance.

In this study, we aimed to analyze the changes in mRNA expression and m^6^A sites in the transcriptome-wide levels of pulmonary artery tissues in monocrotaline (MCT)-induced PAH in rats and controls using methylated RNA immunoprecipitation sequencing (MeRIP-seq) and RNA transcriptome sequencing (RNA-seq) approaches. The functions of differentially expressed genes were further investigated through Gene Ontology (GO) annotation and Kyoto Encyclopedia of Genes and Genomes (KEGG) pathway enrichment. Additionally, the expression of an m^6^A regulator and its role in mediating PASMC biological behaviors was explored to reveal potential molecular mechanisms.

## 2. Materials and Methods

### 2.1. Pulmonary Arterial Hypertension Models and Hemodynamic Measurement

The study was approved by the Animal Ethical and Welfare Committee, the Second Xiangya Hospital, Central South University, China (Approval number 2021489). For PAH induction in male Sprague–Dawley rats, the MCT model was applied. Twelve eight-week-old, weight-matched rats were randomized into two groups: control and MCT. The MCT group was induced via a single intraperitoneal injection of MCT (60 mg·kg^−1^, Sigma-Aldrich, C2401, St. Louis, MO, USA), which produced progressive and severe pulmonary arterial hypertension after 3 weeks, while rats in the control group were injected intraperitoneally with the same volume of saline. Rats were anesthetized, and their right ventricular systolic pressure (RVSP) was measured using pressure transducers. Post-euthanasia, hearts and lungs were rapidly flushed with saline and extracted. Pulmonary artery tissues (including the main pulmonary artery, left and right pulmonary arteries, and lobar pulmonary arteries) were carefully separated on ice within 10 min. The heart’s weight was recorded following removal. The free walls of the right ventricle (RV), left ventricle (LV), and septum (S) were meticulously dissected and weighed to assess the RV/(LV + S) ratio (Fulton index), serving as an indicator of right ventricular hypertrophy.

### 2.2. Histology Staining, Immunohistochemistry, and Immunofluorescence

The right lungs were excised and preserved in situ in an expanded state by infusing them with a 4% paraformaldehyde solution for 24 h. Following this, they were transferred to a 0.2% sodium azide solution prior to paraffin-embedding. The dehydrated tissue was then paraffin-embedded and sliced into 5 mm thick sections. Hematoxylin and eosin staining (from the upper lobe), as well as immunohistochemistry (from the middle lobe) and immunofluorescence (from the lower lobe), was performed on the lung sections, and each of the 12 rats (6 MCT and 6 controls) was used for experiments. Morphometric analyses of pulmonary arteries with an external diameter of 50–100 μm were conducted, and the medial wall thickness was calculated using the following formula: medial thickness (%) = medial wall thickness/external diameter × 100. For quantitative analyses, 20 vessels from each rat were counted, and the averages were calculated. For immunohistochemistry and immunofluorescence, antigen retrieval was performed using citrate antigen retrieval solution (Beyotime, P0081, Shanghai, China), at 80 °C for 20 min. Lung sections were stained with anti- leucine rich pentatricopeptide repeat containing (LRPPRC) (1:100, Proteintech, 21175-1-AP, Wuhan, China) as the primary antibody. For double-labeled immunofluorescence staining, the sections were incubated with primary antibodies against αSMA (1:400, Proteintech, 67735-1-Ig, Wuhan, China) and LRPPRC. Cell nuclei were stained with DAPI (ABP Biosciences, FP026, Beijing, China).

### 2.3. RNA Extraction, MeRIP-Seq and RNA-Seq

Pulmonary artery tissues (n = 6, 3 MCT and 3 controls) were used to extract RNA. Total RNA was isolated using Trizol reagent (Invitrogen, Carlsbad, CA, USA). The concentration and quality of the total RNA were assessed using the Qubit RNA HS Assay and Agilent 2100 Bioanalyzer (Agilent Technology, Palo Alto, CA, USA). M^6^A-modified RNA samples were enriched and sequenced by E-GENE Biotech Inc. (Shenzhen, China). Firstly, 20 μg of total RNA from each sample was fragmented using 10× RNA Fragmentation Buffer (Invitrogen, Carlsbad, CA, USA) in a thermal cycler at 70 °C for 10 min. After fragmentation, the RNA was precipitated with ethanol. Subsequently, 5 μg of an m^6^A antibody (Synaptic Systems, 202 003, Gottingen, Germany) was mixed with pre-washed protein A/G magnetic beads in IP buffer (150 mM NaCl, 10 mM Tris-HCl pH 7.5) and incubated at 4 °C for 2 h [25]. The antibody-bead mixture, with the fragmented RNA, was then suspended in 500 μL of IP reaction mixture and inverted for 4 h at 4 °C. After that, the m^6^A-modified RNA bound to the beads was washed thrice with IP buffer at 4 °C, each for 10 min. Finally, the complexes were incubated at 4 °C for 1 h in 100 μL of m^6^A competitive eluent. Supernatants containing the eluted m^6^A modified RNA were collected and purified with phenol:chloroform:isoamyl alcohol (125:24:1).

The MeRIP libraries of the eluted m^6^A-modified RNA and input RNA samples were constructed using a SMARTer^®^ Stranded Total RNA-Seq Kit version 2 (Takara/Clontech, Otsu, Japan), following the manufacturer’s protocol. The libraries for IP RNA were subjected to fewer than 16 amplification cycles, while those from input RNA underwent fewer than 12 cycles. All libraries were evaluated with an Agilent 2100 Bioanalyzer (Agilent Technology, Palo Alto, CA, USA), and their quantities were confirmed via real-time PCR prior to sequencing. Finally, these libraries were pooled and sequenced using the Illumina Nova platform (Illumina, San Diego, CA, USA). The input libraries of MeRIP-seq were equally used for RNA-seq to analyze and identify differentially expressed genes.

### 2.4. Sequencing Data Analyses

The raw sequencing data were processed using Trimmomatic software (version 0.38, Aachen, Germany). Subsequently, these results were aligned to the human genome reference hg38 using Hisat2 software (version 2.1.0, Baltimore, MD, USA) [26] The R package ExomePeak (version 2.1.2, Suzhou, China) was utilized to identify peaks and discern differential peaks from the refined alignment files [27,28]. Peaks were annotated using ChIPseeker (version 2.16.0, http://bioconductor.org/, accessed on 17 March 2022; Boston, MA, USA) [29]. Peaks with a *p* adjust < 0.05 and fold-change (FC) ≥ 1.5 were identified as significantly different. The gene expression calculation was performed using StringTie software (version 2.2.0, http://ccb.jhu.edu/software/stringtie/, accessed on 19 March 2022; Baltimore, MD, USA). The gene expression profiling was based on the number of reads. The transcripts per million (TPM) mapped reads values were used to estimate the expressed values and transcript levels. Genes with an adjusted *p*-value (*p* adj) < 0.05 and absolute FC ≥ 1.5 were assigned as differentially expressed genes (DEGs) identified by the DESeq2 R package [30]. The KEGG and GO analyses were performed using cluster profiler software (version 4.2.2, https://www.bioconductor.org/, accessed on 23 October 2023) to predict linked biological functions and pathways. The identification of motifs for m^6^A-modified regions was performed using HOMER (version 4.11, http://homer.ucsd.edu/, accessed on 1 October 2023) [31]. The protein–protein interaction (PPI) network construct was performed using STRING (version 11.5, https://string-db.org, accessed on 23 October 2023) and Metasacpe (version 3.5, https://metascape.org, accessed on 23 October 2023) databases. The m^6^A patterns of gene methylated sites were visualized using Integrative Genomics Viewer software (version 2.15.4, https://software.broadinstitute.org/, accessed on 7 October 2023) [32].

### 2.5. Data Collection and DEGs Identification

GEO is a public functional genomic data repository, and we used “pulmonary hypertension” and “microarray” as keywords to search for newly submitted transcriptomic datasets in the last 5 years. The search focused on datasets from the *Homo sapiens* species, with lung tissue samples, and targeting the disease IPAH. Finally, two datasets, GSE113439 and GSE130391, were selected for further analysis. The information about these datasets is provided in Table 1. DEG analysis was performed by using GEO2R (https://www.ncbi.nlm.nih.gov/geo/geo2r, accessed on 24 October 2023). Genes exhibiting a |log2 fold-change (FC)| value of ≥0.585 and an adjusted *p*-value of <0.05 were identified as DEGs from each dataset, with *p*-values adjusted using the false discovery rate (FDR) method. The intersecting set of genes from both datasets was derived using the R package “ggven”.

### 2.6. Protein Isolation and Western Blots

Left lung tissues and cell samples were homogenized in lysis buffer containing a complete protease inhibitor cocktail (Roche, Basel, Switzerland). Proteins were run on 10% sodium dodecyl sulfate-polyacrylamide gels, followed by transfer onto nitrocellulose membranes (Millipore, Billerica, MA, USA). After blocking, membranes were probed with one of the following primary antibodies overnight at 4 °C: LRPPRC (1:1000, Proteintech, 21175-1-AP, Wuhan, China), PCNA (1:1000, Proteintech, 10205-2-AP, Wuhan, China), and α-Tubulin (1:1000, Proteintech, 11224-1-AP, Wuhan, China). Secondary antibodies were purchased from Proteintech and applied at a 1:5000 dilution. Final detections of the proteins were performed using a ChemiDoc^TM^ MP imaging system (Bio-Rad, Hercules, CA, USA).

### 2.7. Quantitative Real-Time PCR

Left lung tissues and cell samples were extracted with Trizol reagent (Invitrogen, Carlsbad, CA, USA) to gain total RNA. This RNA was then utilized for cDNA synthesis via the SuperScript™ First-Strand cDNA Synthesis Kit User Manual (GeneCopoeia^TM^, Guangzhou, China). Real-time PCR was conducted using the BlazeTaq^TM^ SYBR Green qPCR Mix (GeneCopoeia^TM^, Guangzhou, China) and QuantStudio^TM^ Real-Time PCR System (Thermo Fisher Scientific, Waltham, MA, USA) following the manufacturer’s guidelines. The primer sequences employed are listed in Table 2.

### 2.8. Primary Rat and Human PASMC Culture and siRNA Knockdown of Lrpprc

Rats were euthanized under anesthesia, and their lung tissues were promptly dissected. After peeling off connective tissue, the pulmonary artery was dissected and cut into small pieces. Cells were digested in HBSS with 1 mg/mL collagenase I (Sigma-Aldrich, St. Louis, MO, USA) at 37 °C for a duration of 20 min. Lysates of tissues were filtered through a 0.45 μm cell strainer and centrifuged for collecting cells. After culture in DMEM/F12 with 20% fetal bovine serum (FBS) to reach 80% confluency, cell purity was confirmed via immunofluorescence with αSMA (1:400, ProteinTech, 67735-1-Ig, Wuhan, China), CD31 (1:100, ProteinTech, 11265-1-AP, Wuhan, China), and Vimentin (1:100, ProteinTech, 10366-1-AP, Wuhan, China). Human PASMCs were purchased from Abiowell (AW-YCH033, Changsha, China) and cultured the same as rat PASMCs. PASMCs were used in passages 3–5. To achieve gene silencing in PASMCs, cells were transfected with control siRNA and si-*Lrpprc* or si-*CENPF* in antibiotic-free medium for 48 h. The si-RNA for *Lrpprc* was synthesized by Ribobio (Guangzhou, China) with the sequence 5′-GGAGCAAGATAACAGAATT-3′. The sequence of si-*CENPF* was 5′-GAAUGAUUCACUUAAGGA-3′.

### 2.9. Proliferation Assays

PASMC proliferation was determined during stimulation with 30 ng/mL Platelet-Derived Growth Factor-BB (PDGF-BB) (Peprotech, Rocky Hill, NJ, USA) after being starved in 0.5% FBS-supplemented medium for 24 h. Cell proliferation and apoptosis assays were respectively performed using an EdU Cell Proliferation Kit with Alexa Fluor 555 (Epizyme, CX003, Shanghai, China) according to the manufacturer’s protocols.

### 2.10. Statistical Analysis

Data are presented as the mean ± standard error of the mean (SEM). Student *t*-tests and one-way ANOVA were used to compare two and multiple groups. GraphPad Prism (version 7.00, La Jolla, CA, USA) was applied to perform the statistical analysis. A two-sided *p*-value of <0.05 was considered statistically significant.

## 3. Results

### 3.1. Basic Characteristics of m^6^A in PAH Rat Models

**Figure 1:**
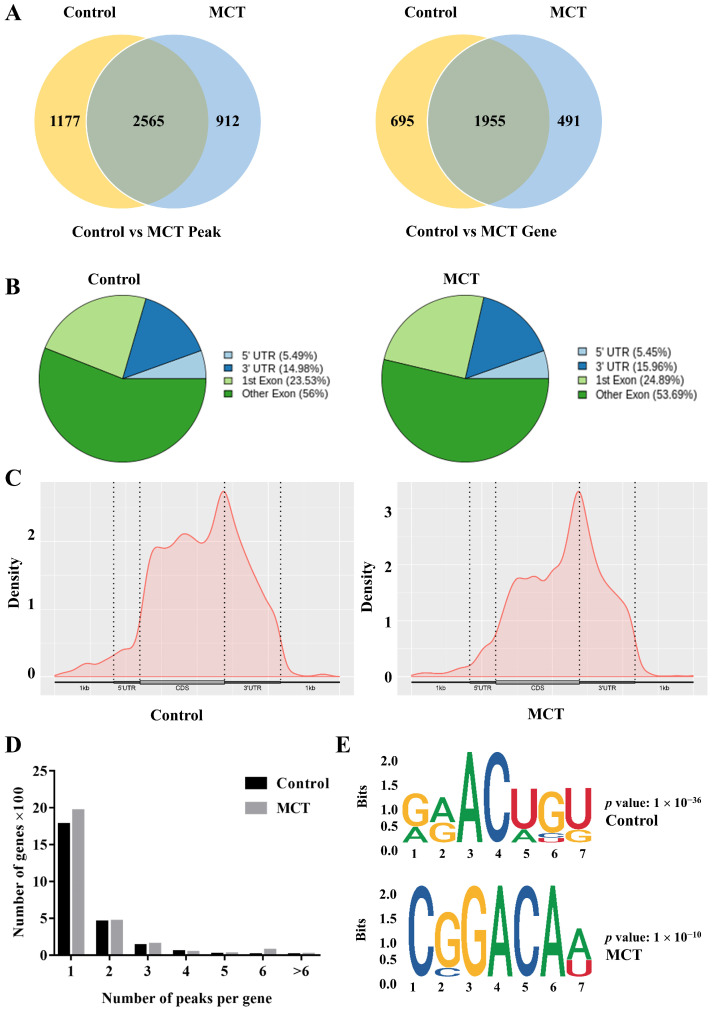
Basic characteristics of m^6^A in PAH rat models. Tissues were from pulmonary arteries, n = 3 each. (**A**) Venn diagram showing the overlap of m^6^A peaks (**left**) and genes (**right**) in the two groups. (**B**) Pie chart showing the percentage of m^6^A peaks in four non-overlapping segments of transcripts. (**C**) Density curve showing the distribution of m^6^A peaks across the transcripts. The transcript is divided into three parts, namely 5′ UTR, CDS, and 3′ UTR. (**D**) The proportion of genes harboring different numbers of m^6^A peaks in the two groups. (**E**) The RRACH motifs are in two groups. m^6^A: N6-methyladenosine; PAH: pulmonary arterial hypertension; MCT: monocrotaline; UTR: untranslated region; CDS: coding sequence.

Because obtaining lung tissue samples from PAH patients is difficult, we established the MCT-induced PAH rat model for further experiments (Supplementary Figure S1). To determine the changes in the RNA methylation pattern between normal and hypertensive pulmonary arteries, the percentage of m^6^A was examined in pulmonary artery tissues of established PAH rat models induced via an MCT injection. As shown in Figure 1A, MeRIP-seq analysis identified 3742 m^6^A peaks within 2560 genes in the control and 3477 m^6^A peaks within 2246 genes in the MCT group. Of those, 2565 peaks within 1955 genes (55.1% of all peaks in the control and MCT groups) overlapped, which indicated that the pulmonary artery tissues had abundant m^6^A levels. To reveal the preferential distribution of m^6^A in the whole transcriptomes of the two groups, we systematically classified these m^6^A sites into three transcript regions: 5′untranslated region (5′ UTR), the coding sequence (CDS, including the first exon and other exons), and 3′ UTR in the entire transcriptomes (Figure 1B). The m^6^A peak density climbed rapidly between the 5′ UTR and the start codon, gradually increasing in the CDS region. Interestingly, the density rocketed to the highest value between the CDS and 3′ UTR. In the 3′ UTR segment, the density decreased rapidly to a low level (Figure 1B,C). Notably, most genes exhibited one or two m^6^A peaks, while a smaller subset displayed three or more m^6^A peaks in both the control and MCT groups (Figure 1D). M^6^A predominantly occurs on adenine within the RRACH (R = A or G; H = A, C, or U) sequence motif, particularly near the start of the 3′ UTR, close to the translation stop codon [35,36]. Subsequently, we employed the HOMER software to assess whether the m^6^A classical consensus sequence was identified in the m^6^A detection process. As shown in Figure 1E, m^6^A motif analysis showed that the classical RRACH motifs were detected in the region of m^6^A peaks in both the control and MCT groups. Together, these findings validate our data’s accuracy and illustrate the m^6^A patterns in PAH transcripts of MCT and control rats.

### 3.2. Differential m^6^A Analysis in PAH

**Figure 2:**
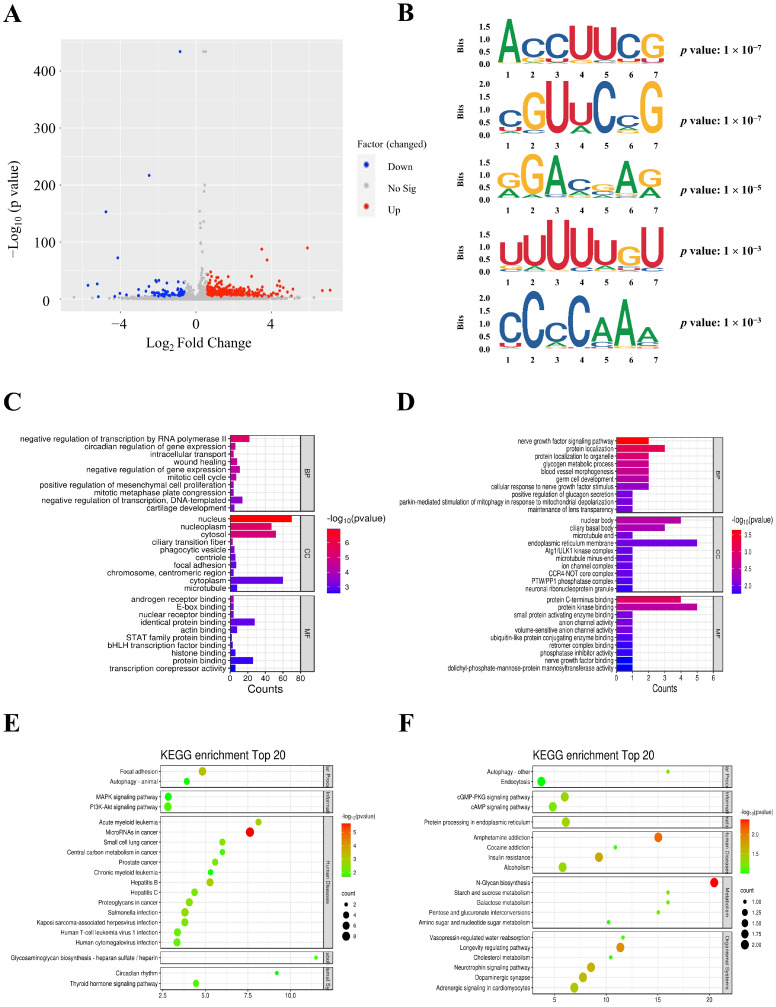
Differential m^6^A analysis in PAH. (**A**) A volcano plot of differentially expressed m^6^A peaks in theMCT group relative to the control (|log2 FC| > 0.585, *p* < 0.05). (**B**) The top five motifs enriched across the differential m^6^A peaks. (**C**) GO analysis of upregulated m^6^A-tagged transcripts with differential m^6^A. (**D**) GO analysis of downregulated m^6^A-tagged transcripts with differential m^6^A. (**E**) KEGG analysis of upregulated m^6^A-tagged transcripts with differential m^6^A modification. (**F**) KEGG analysis of downregulated m^6^A-tagged transcripts with differential m^6^A. FC: fold change; GO: gene ontology; KEGG: kyoto encyclopedia of genes and genomes.

To further clarify the changes in m^6^A linked with PAH, an analysis focusing on the differences in the m^6^A level analysis was conducted. Setting the statistical standard as *p* adjust < 0.05 and FC ≥ 1.5, a total of 218 differentially m^6^A peaks within 209 genes between the MCT and control groups were identified via MeRIP-seq analysis (Supplementary Table S1 and Table 3). Among them, 45 m^6^A sites in 45 genes exhibited hypomethylation, and 173 sites in 164 genes showed hypermethylation (Figure 2A). The analysis of differentially methylated m^6^A peaks showed that most were concentrated in the CDS region, with the highest density at the boundaries between the CDS and 3′ UTR (Supplementary Figure S2). The top five motifs identified among the differential m^6^A peaks are shown in Figure 2B.

Genes with significantly altered m^6^A peaks were selected for GO and KEGG enrichment analyses to reveal the pathways of m^6^A in PAH. GO analysis showed that in the molecular functions (MF) category, both up- and down-methylated m^6^A genes were mainly enriched in regulating RNA transcription, including E-box binding, nuclear receptor binding, and protein C-terminus binding. In the biological processes (BPs) category, up-methylated m^6^A genes were enriched significantly in gene expression, and down-methylated m^6^A genes mainly participated in protein localization. Meanwhile, the differentially m^6^A genes were mainly located in the nucleus in the cellular components (CCs) category (Figure 2C,D). Enriched KEGG pathways indicated that the up-methylated m^6^A genes were mostly concentrated in the focal adhesion, MAPK, and PI3K/AKT signaling pathways, while the down-methylated sites were enriched in the N-glycan biosynthesis, cGMP/PKG, and cAMP signaling pathways (Figure 2E,F).

### 3.3. Transcriptional Profile of m^6^A-Modified Genes

**Figure 3:**
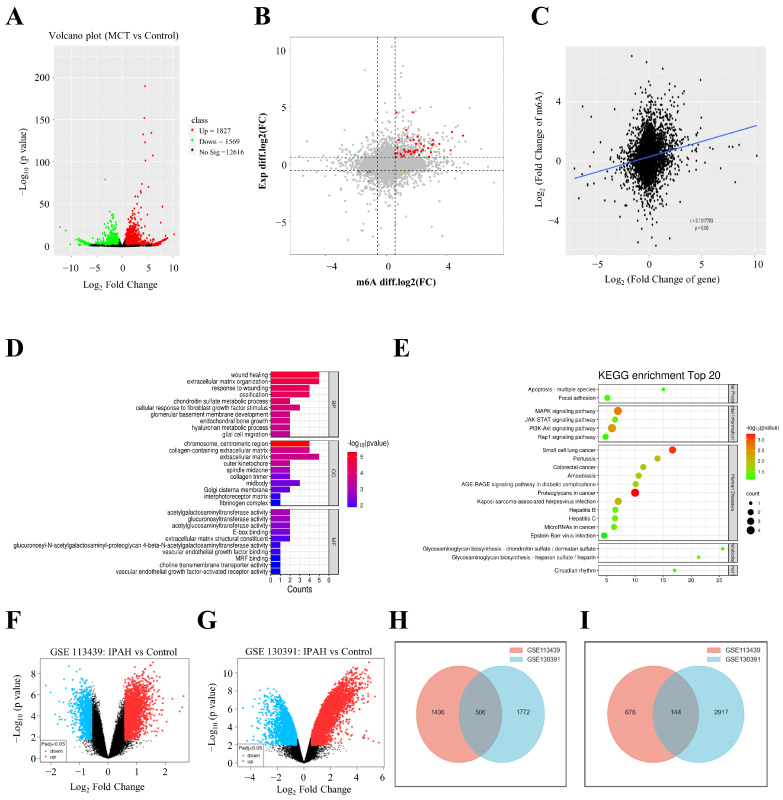
Transcriptional profile of m^6^A-modified genes. (**A**) A volcano plot of differentially expressed genes. (**B**) The four-quadrant graph of differentially expressed genes with significantly changed peaks. (**C**) Positive correlation between overall m^6^A and the mRNA expression level (r = 0.1517793, *p* < 0.05). (**D**,**E**) GO and KEGG analyses of differentially expressed genes with significantly changed peaks, respectively. (**F**,**G**) Volcano plots of DEG distributions for the two datasets. A red spot represents an upregulated DEG, a blue spot represents a downregulated DEG, and a black spot represents a non-DEG. (**H**) DEGs are upregulated simultaneously in both datasets. (**I**) DEGs are downregulated simultaneously in both datasets. DEG: differentially expressed gene.

We further identified the transcriptional level changes in differential m^6^A-modified genes by integrating RNA-seq and MeRIP-seq datasets. The volcano plot of MCT vs. control was generated using the RNA-seq approach with a total of 1827 upregulated mRNAs and 1569 downregulated mRNAs (*p* adjust < 0.05, FC ≥ 1.5; Supplementary Table S2 and Figure 3A). A four-quadrant graph showed the fold-changes in differential m^6^A peaks and RNAs (Supplementary Table S3 and Figure 3B). A total of 37 hypermethylated genes were upregulated (marked red), one hypermethylated gene was downregulated (marked yellow), one hypomethylated gene was upregulated (marked cyan), and three hypomethylated genes were downregulated (marked blue) in the two groups. Our results further showed a positive correlation between m^6^A abundance and the gene expression level in MCT and control samples (Figure 3C). Enriched pathways of these total 42 genes were further demonstrated through GO and KEGG analyses. The BP and MF analyses were mainly associated with ECM structure and organization, and the CC analysis significantly enriched the chromosome, centromeric region, and extracellular matrix (Figure 3D). GO analysis suggested that m^6^A may be involved in PAH by acting on the ECM. KEGG analysis showed these genes were mainly enriched in the MAPK and PI3K/AKT signaling pathways (Figure 3E). To explore the changes in these 42 genes in PAH patients, we selected two gene expression datasets, GSE113439 and GSE130391, for this study. Data from IPAH (idiopathic PAH) patients and healthy individuals were selected, normalized, and annotated. This preprocessed dataset was then analyzed using the GEO2R platform to identify differentially expressed genes (DEGs). In the GSE113439 dataset, we found 2732 DEGs (Supplementary Table S4) and 5339 DEGs in the GSE130391 dataset (Supplementary Table S5). We used the Venny 2.1 platform to determine the intersecting DEGs between these datasets, identifying 506 upregulated DEGs (Supplementary Table S6) and 144 downregulated DEGs (Supplementary Table S7). Volcano and Venn diagrams for the DEGs in each dataset are presented in Figure 3F–I. From these findings, we noted that Centromere Protein F (*CENPF*), Marker of proliferation Ki-67 (*MKI67*), Poly (ADP-Ribose) Polymerase Family Member 14 (*PARP14*), and Transcriptional Repressor GATA Binding 1 (*TRPS1*) were consistently upregulated in IPAH patients, while 38 other genes showed no significant statistical difference (Table 4).

### 3.4. PPI Network Establishment and Candidate Gene Identification

**Figure 4:**
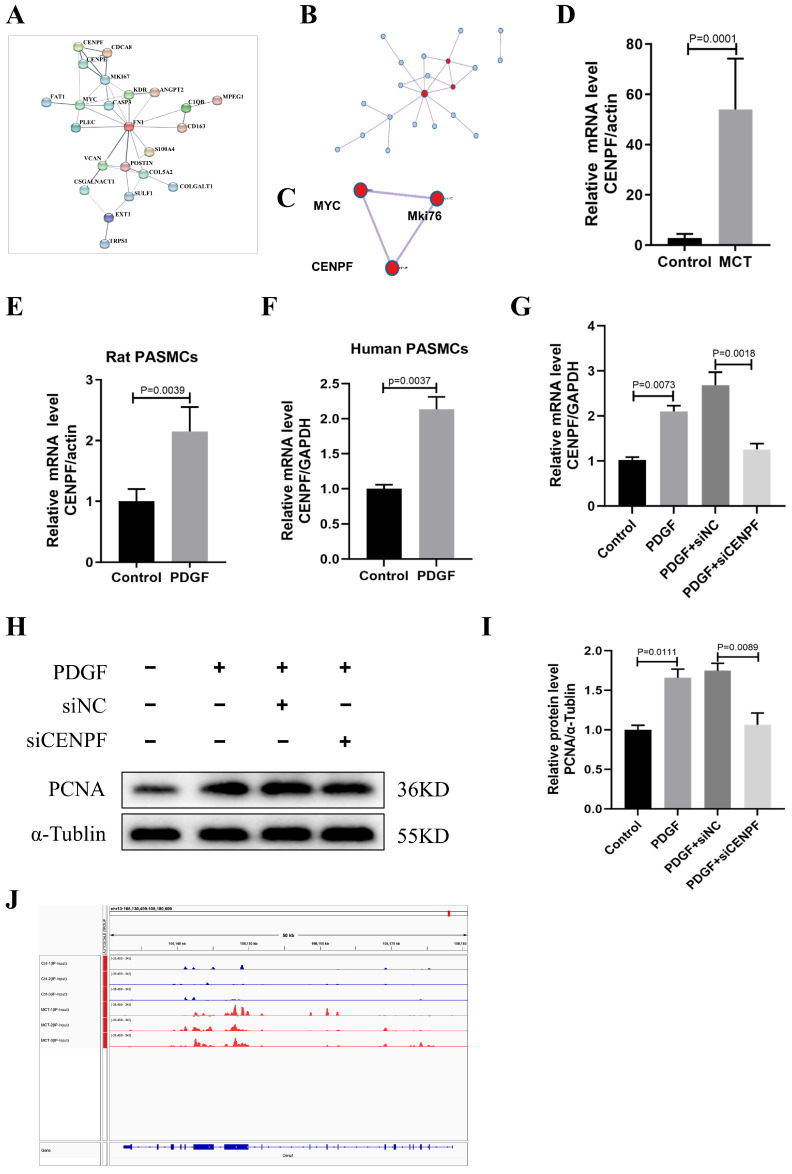
PPI network establishment and candidate gene identification. (**A**–**C**) The hub network was constructed based on the genes presented above. (**D**) Relative mRNA expression of *Cenpf* in the lung tissues of MCT and control groups (n = 6 each), data represent the mean ± SEM, and a Student *t*-test was used to compare the two groups. (**E**) Relative mRNA expression of *Cenpf* in rat PASMCs under PDGF-BB stimulation (30 ng/mL) normalized to the α-Tublin internal control (n = 3 each). (**F**) Relative mRNA expression of *CENPF* in human PASMCs under PDGF-BB stimulation (30 ng/mL) normalized to the *GAPDH* internal control (n = 3 each). (**G**) Human PASMCs were transfected with siRNA for 48 h before treatment with PDGF-BB (30 ng/mL) for another 24 h. Relative mRNA expression of *CENPF* levels (n = 3 each). (**H**,**I**) Representative western blots and quantification of PCNA levels normalized to the α-Tubulin internal control (n = 3 each). (**J**) The level of m^6^A on *Cenpf* mRNA transcripts observed via IGV. PPI: protein–protein interaction; SEM: standard error of mean; PASMCs: pulmonary artery smooth muscle cells; PDGF-BB: platelet-derived growth factor-BB.

To construct the PPI networks, the above 42 genes were incorporated based on the STRING database and showed extensive interactions (Figure 4A). Next, we used the Metascape database to determine the hub genes in the PPI networks. As shown in Figure 4B,C, MYC Proto-Oncogene (*MYC*), *MKI67*, and *CENPF* were identified as hub genes. *CENPF* was selected with relatively high expression among the three central genes for further verification. In the subsequent analysis via qRT-PCR, the expression of *Cenpf* was significantly increased in the MCT groups compared with the control (Figure 4D). Since CENPF was enriched in the mitotic cell cycle process linked with proliferation in GO analyses, we isolated and determined primary rat PASMCs (Supplementary Figure S3) and exposed them to PDGF-BB, a cytokine suggested to be a crucial factor for PASMC proliferation during the advancement of PAH. As shown in Figure 4E, the upregulation of *Cenpf* was observed upon PDGF-BB stimulation in PASMCs. Additionally, we used human primary PASMCs and exposed them to PDGF-BB. The result was consistent with that in rats (Figure 4F). To further explore the role of CENPF in pulmonary vascular remodeling, we silenced *CENPF* in the human PASMCs using siRNA treatment with PDGF-BB stimulation. The results showed that decreased CENPF inhibits PASMC proliferation (Figure 4G–I). Figure 4J showed the peak of *Cenpf* in the control and MCT groups. These results indicated that CENPF was increased in PAH and can be regulated by m^6^A changes.

### 3.5. LRPPRC Is Decreased in the Pulmonary Arteries of PAH

**Figure 5:**
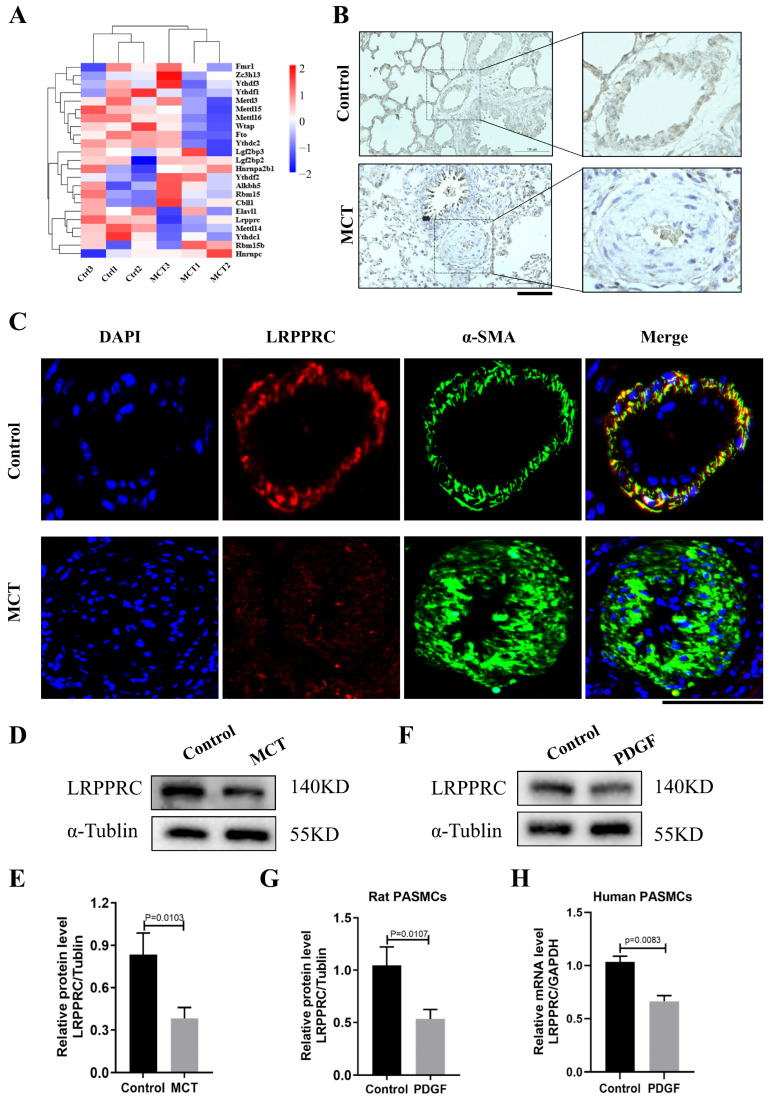
LRPPRC was decreased in the pulmonary arteries of PAH. (**A**) The heatmap for the expression of 23 m^6^A regulators in the two groups. (**B**) Immunohistochemical staining of LRPPRC in lung tissues (n = 6 each). Bar = 100 µm (**left** panel). Enlarged pulmonary artery (**right** panel). (**C**) Immunofluorescence of LRPPRC in lung tissues. Vascular staining image (green), objective protein staining image (red), nuclei staining image (blue), and merged images (orange) (n = 6 each). Bar = 50 µm. (**D**,**E**) Representative Western blots and quantification of LRPPRC and α-Tubulin in lung tissues of MCT and control groups (n = 6 each); data represent the mean ± SEM and a Student *t*-test was used to compare the two groups. (**F**,**G**) Representative Western blots and quantification of LRPPRC levels in rat PASMCs under PDGF-BB stimulation (30 ng/mL) normalized to the α-Tubulin internal control (n = 3 each). (**H**) Relative mRNA expression of *LRPPRC* in human PASMCs under PDGF-BB stimulation (30 ng/mL) normalized to the *GAPDH* internal control (n = 3 each).

The changed m^6^A levels in the pulmonary arteries in PAH could be due to the dysregulation of methylation-related enzymes. The heatmap presented the mRNA expression levels of 23 previously reported m^6^A-related regulators, including writers, readers, and erasers (Figure 5A). Transcriptional levels of six regulators were shown to be significantly changed as follows (*p* < 0.05, seen in Table 5): four downregulated (*Mettl14*, *Mettl15*, *Mettl16*, and *Lrpprc*) and two upregulated (*Hnrnpc* and *Ythdf2*). LRPPRC was selected for further study due to its remarkably decreased expression level, based on RNA-seq, in PAH models. To elucidate LRPPRC’s expression and distribution in pulmonary arteries, immunohistochemistry staining and immunofluorescence were performed on rat lung tissues. In the control, LRPPRC was mostly localized to the PASMC layer in small pulmonary arteries, whereas it was rarely expressed in small pulmonary arteries of MCT rats compared to the normal control (Figure 5B,C). Subsequent Western blotting showed that the LRPPRC protein level in the MCT group showed a similar decreased tendency compared to the control group (Figure 5D,E). As shown in Figure 5F,G, the expression of LRPPRC was downregulated upon PDGF-BB stimulation in rat PASMCs. Subsequently, we detected decreased *LRPPRC* mRNA levels in PDGF-BB-stimulated human PASMCs (Figure 5H). Taken together, these results demonstrate that as an m^6^A reader, the reduced level of LRPPRC may possibly be involved in the development of PAH.

### 3.6. Downregulation of LRPPRC Promotes PASMC Proliferation and Cenpf Expression

**Figure 6:**
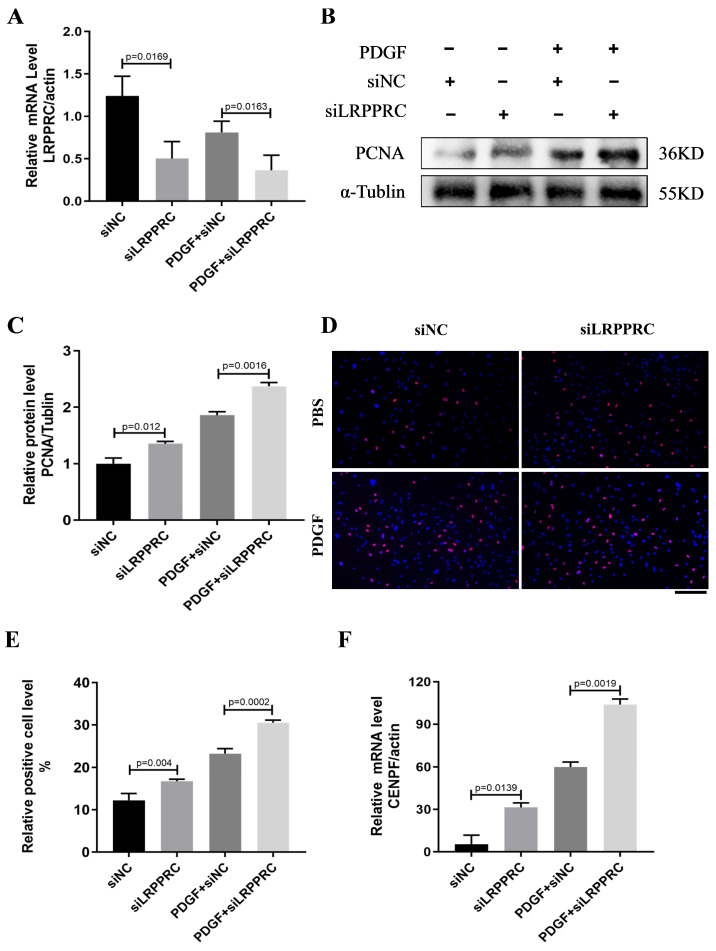
Downregulation of LRPPRC promotes PASMC proliferation and *Cenpf* expression. (**A**) PASMCs were transfected with siRNA for 48 h before treatment with PDGF-BB (30 ng/mL) for another 24 h. Relative mRNA expression of *Lrpprc* levels (n = 3 each). (**B**,**C**) Representative Western blots and quantification of PCNA levels normalized to the α-Tubulin internal control (n = 3 each). (**D**) Cell proliferative ability was determined via an EdU assay in si-*Lrpprc*-PASMCs with PDGF-BB stimulation. Bar = 100 µm. Objective cells (red), and (**E**) calculation of EdU-stained cell rates. (**F**) Relative mRNA expression of *Cenpf* levels (n = 3 each).

Pulmonary vascular remodeling mainly manifests as uncontrolled proliferation and apoptosis resistance in PASMCs [3]. To investigate whether LRPPRC is essential for the vascular remodeling of PAH, we silenced *Lrpprc* in the PASMCs using siRNA treatment with or without PDGF-BB stimulation (Figure 6A). Enhanced PASMC proliferation induced by downregulated *Lrpprc* was observed based on Western blots (Figure 6B,C). A cell proliferation assay showed a similar increase in the number of proliferating cells (marked red, Supplemental Table S8, Figure 6D,E). To explore whether LRPPRC is a potential reader for *Cenpf*, we detected *Cenpf* mRNA expression after *Lrpprc* knockdown. As shown in Figure 6F, the downregulation of LRPPRC significantly increased the *Cenpf* mRNA level based on qRT-PCR. These findings demonstrated that LRPPRC plays a negative role in PASMC proliferation and the transcriptional level of *Cenpf* expression.

## 4. Discussion

Unlike genetic mutations, many epigenetic modifications are pharmacologically catalyzed by reversible enzymatic reactions, making them attractive therapeutic targets of PAH. Previous studies have identified decreased m^6^A levels in circRNAs under hypoxia-mediated pulmonary hypertension [22]. Furthermore, elevated m^6^A levels were observed in the peripheral blood and lungs of IPAH patients, as well as experimental PH models [37,38]. However, no studies have reported on how the m^6^A modification profile is changed in pulmonary artery tissues of PAH. Here, we showed a panoramic view of m^6^A-modified RNA in pulmonary artery tissues from rats with MCT-induced PAH. A further integrated analysis of our MeRIP-seq and RNA-seq data provided transcriptomic profiling for an in-depth investigation of m^6^A in PAH. Then, we identified that the hypermethylated gene *Cenpf* was significantly increased in PAH pulmonary arteries and proliferative PASMCs. Furthermore, this study was the first to demonstrate that the differentially expressed m^6^A reader LRPPRC inhibits PASMC proliferation and downregulates the mRNA expression of *Cenpf*.

Altered m^6^A implies its potential role as a modifier in PAH pathogenesis. In this study, we found 209 genes with significant changes in m^6^A following PAH. To investigate the biological function influenced by RNA m^6^A, GO and KEGG pathway enrichment analyses were carried out. In the top 30 GO terms, genes with differential m^6^A were enriched mainly in the cell cycle, RNA transcription, and protein localization, which indicates that m^6^A plays a critical and extensive role in the cellular signaling activities of PAH. Consistent with the sequencing results in rat MCT-PAH lung tissues by Zeng et al., our KEGG analysis demonstrated that the MAPK and PI3K/AKT signaling pathways were enriched [39]. In our subsequent combined analysis, we identified 42 mRNAs present in the differentially m^6^A-modified mRNA group and the differentially expressed mRNA group. Interestingly, the GO enrichment showed that the most involved process was ECM, and the KEGG main pathways were MAPK and PI3K/AKT. Accumulating evidence shows that ECM remodeling occurs in the early phase of the vascular remodeling process, before the intimal and medial thickness, implying that ECM might be a driving factor, rather than a result of distal pulmonary vascular remodeling [40]. ECM remodeling promotes pulmonary vascular cells’ biological functions by activating various signaling pathways, such as the classical downstream PI3K/AKT signaling, and regulating abnormal PASMC proliferation, migration, and phenotypic transitions [40]. Therefore, we propose that m^6^A might be intimately associated with the development and progression of pulmonary vascular remodeling, which provides new insight into the strategies for reversing PAH.

Three important hub genes, *MYC*, MKI67, and *CENPF*, were identified as upregulated through a bioinformatic analysis and screening. CENPF is crucial for cell mitosis, being concentrated in the G2/M phase of the cell cycle and utilized during the mitosis process [41]. The aberrant expression of CENPF has been found in multiple malignancies, and accumulating literature has asserted that CENPF is closely related to cell proliferation and tumorigenesis, but little is known about it in PAH [42,43]. Our real-time PCR results revealed an increased transcriptional level of *Cenpf* in the pulmonary arteries of PAH rats and PASMCs, confirming its participation in the pathogenesis of PAH.

The m^6^A “reader” proteins selectively recognize m^6^A sites and mediate the degradation of the m^6^A-modified mRNA, indicating a potential inverse correlation between the degree of m^6^A and the transcriptional level [44]. Nonetheless, this finding is somewhat at variance with our current observations based on the pulmonary arteries of PAH rats, which showed that many hypermethylated mRNAs also have increased transcriptional levels. These variations may result from distinct methodologies, biological species, or tissue samples. The results also suggest that different tissues may have unique features at m^6^A sites, pointing to a regulatory function of m^6^A in gene expression.

For LRPPRC—the new differentially expressed m^6^A reader in this study—we subsequently verified its expression via Western blotting and immunohistochemistry staining. Finally, we first identified that the reader LRPPRC could be a potent m^6^A-modified reader protein with a markedly downregulated change in MCT-PAH rat PASMC layers of pulmonary arteries. LRPPRC is an RNA-binding protein that regulates gene translation, RNA polyadenylation, and RNA stability [45,46]. It is involved in multiple pathophysiological processes, including autophagy, glycolysis, and oxidative stress [47,48,49]. LRPPRC was detected as upregulated in lung and gastric adenocarcinoma, leading to apoptosis resistance and invasive activity in these cancer cells via its overexpression in vitro [50]. However, it has also been reported that LRPPRC protein levels are reduced in diethylnitrosamine-induced mouse hepatocellular carcinoma tissues, and the liver-specific deletion of *Lrpprc* in mice resulted in a higher likelihood of developing hepatocellular carcinoma and a shorter survival time [49]. These reports suggest that the expression profile of LRPPRC is tissue-type-dependent. In our study, the mRNA and protein levels of LRPPRC were both decreased in the MCT group compared with the control group. To explore its biological functions, it was further demonstrated that the reduction in LRPPRC led to a proliferative effect in PASMCs. The cancer-like patho-mechanisms of unexplained proliferation in PASMCs are consistent with the PAH phenotype. Moreover, we found that the elevated transcriptional level of *Cenpf* was observed by knocking down LRPPRC. These findings suggest that in PAH, the expression of LRPPRC was decreased and the knockdown of LRPPRC could facilitate proliferation and *Cenpf* expression in PASMCs. Thus, we hypothesize LRPPRC may mediate proliferative functions by coordinating the modification of m^6^A-targeted *CENPF* in PAH.

This study presents some possible limitations. Initially, m^6^A is a dynamic and reversible process, so the modification level of genes would change dynamically during the occurrence and development of PAH. In this study, we only chose one time point to explore the change in m^6^A in PAH. Second, in this study, we only used the MCT-PAH model to validate gene expression. In addition, our study showed that the downregulation of LRPPRC and CENPF can alter the biological behavior of PASMCs, while the effect of gene overexpression and its exact mechanisms are still unclear. Based on these potential limitations, our further study will design longitudinal studies in vivo and in vitro to provide a more comprehensive understanding of the temporal dynamics of PAH and use tissues from different animal models and human samples for further validation. Moreover, constructing transgenic animals to further explore the function and molecular mechanism of relative genes will enrich our future research direction.

## 5. Conclusions

In conclusion, this study suggests that m^6^A plays a key role in the development and progression of PAH. Decreased expression of the m^6^A reader LRPPRC promotes the proliferation of PASMCs and increases the mRNA level of *Cenpf*. Therefore, a better understanding of m^6^A may facilitate novel potential strategies for future PAH treatment.

## Figures and Tables

**Table 1:** Dataset detailed information.

References	Sample	GEO	Platform	IPAH	Control
Mura, M. et al. [33].	Lung tissue	GSE113439	GPL6244	6	11
Halliday, S. et al. [34].	Lung tissue	GSE130391	GPL570	4	4

**Table 2:** Primers used for quantitative real-time PCR.

Gene (Species)	Primer	Gene Sequence
*β-actin* (rat)	Forward	ACATCCGTAAAGACCTCTATGCC
	Reverse	TACTCCTGCTTGCTGATCCAC
*Cenpf* (rat)	Forward	TTTGTGAGGAGCTCGGCG
	Reverse	AGCATCTAGAAGTAAACTGAGCG
*Lrpprc* (rat)	Forward	TGGTCTTCATCAACAACATTGCTCTG
	Reverse	GGCTCCATCTGCTCCTCTATCACT
*GAPDH* (human)	Forward	CTTCGCTCTCTGCTCCTCCTGTTCG
	Reverse	ACCAGGCGCCCAATACGACCAAAT
*CENPF* (human)	Forward	CGTCCCCGAGAGCAAGTTTA
	Reverse	GTAGGCAGCCCTTCTTTCCA
*LRPPRC* (human)	Forward	GACGTTCGAGCAATGGCAG
	Reverse	CTCAGTAGTCCTCCGGCCAC
*Cenpf*: centromere protein F; *Lrpprc*: leucine rich pentatricopeptide repeat containing.		

**Table 3:** Total numbers of differentially methylated m^6^A peaks and associated genes.

Pair	Hypermethylated Peaks	Hypermethylated Genes	Hypomethylated Peaks	Hypomethylated Genes
MCT-Control	173	164	45	45

**Table 4:** The mRNA expression levels (Log2(FC)) of genes in databases.

Gene ID	DGEs	GSE113439	GSE130391
*CENPF*	2.46979	0.775	0.945
*MKI67*	2.39000	0.747	1.271
*PARP14*	0.81945	0.886	1.321
*TRPS1*	0.64099	0.750	1.860

*MKI67*: marker of proliferation Ki-67; *PARP14*: poly (ADP-Ribose) polymerase family member 14; *TRPS1*: transcriptional repressor GATA binding 1.

**Table 5:** The mRNA expression levels of m^6^A regulators.

Gene ID	Regulation	Base Mean	Log2(FC)	*p*
*Mettl14*	Writer	866.635	−0.475	0.008
*Mettl15*	Writer	97.199	−0.993	0.020
*Mettl16*	Writer	463.047	−0.652	0.007
*Lrpprc*	Reader	1884.530	−0.820	0.002
*Hnrnpc*	Reader	399.525	1.345	0.021
*Ythdf2*	Reader	688.647	0.494	0.043

*Mettl14*: methyltransferase 14; *Mettl15*: methyltransferase 15; *Mettl16*: methyltransferase 16; *Hnrnpc*: heterogeneous nuclear ribonucleoprotein C; *Ythdf2*: YTH N6-methyladenosine RNA binding protein F2.

## Data Availability

All data generated or analyzed during this study are included in this published article and its supplementary information files.

## Supplementary Materials

The following supporting information can be downloaded at: https://www.mdpi.com/article/10.3390/biomedicines12020364/s1, Figure S1: PAH rat model validation; Figure S2: Differential m^6^A analysis in PAH; Figure S3: PPI network establishment and candidate gene identification. Table S1: Differentially methylated m^6^A peaks and associated genes. Table S2: Differentially ex-pressed genes in rat pulmonary arteries. Table S3: Combination of differentially expressed genes and differentially methylated m^6^A peaks. Table S4: Differentially expressed genes in GSE113439 dataset. Table S5: Differentially expressed genes in GSE130391 dataset. Table S6: Intersecting upregulated DEGs in two GEO datasets. Table S7: Intersecting downregulated DEGs in two GEO datasets. Table S8: EdU cell numbers.
