# Supplementary material for: Transcriptome-Wide N6-Methyladenosine Alternations in Pulmonary Arteries of Monocrotaline-Induced Pulmonary Arterial Hypertension in Rats and Novel Therapeutic Targets
Source: Biomedicines. 2024 Feb 4;12(2):364. doi: 10.3390/biomedicines12020364 (PMC10886831; doi:10.3390/biomedicines12020364)
Supplement: Supplementary file 1 [file biomedicines-12-00364-s001.zip › SUPPLEMENTAL INFORMATION.pdf]

SUPPLEMENTAL INFORMATION

Transcriptome-Wide N6-Methyladenosine Alternations in Pulmonary Arteries of Monocrotaline-Induced Pulmonary Arterial Hypertension in Rats and Novel Therapeutic Targets

Yilu Feng 1,2,3, Zaixin Yu 1, Mi Tang 4, Jiang Li 2,3, Baohua Peng 1,

Mukamengjiang Juaiti 1, Yiyang Tang 1, Benhui Liang 1, Mingqi Ouyang 2,3,

Qingqing Liu 5 and Jie Song 2,3,*

<u>**Inventory of Supplemental Information**</u>

Supplemental Figure S2: Linked to Figure 2

Supplemental Figure S3: Linked to Figure 4

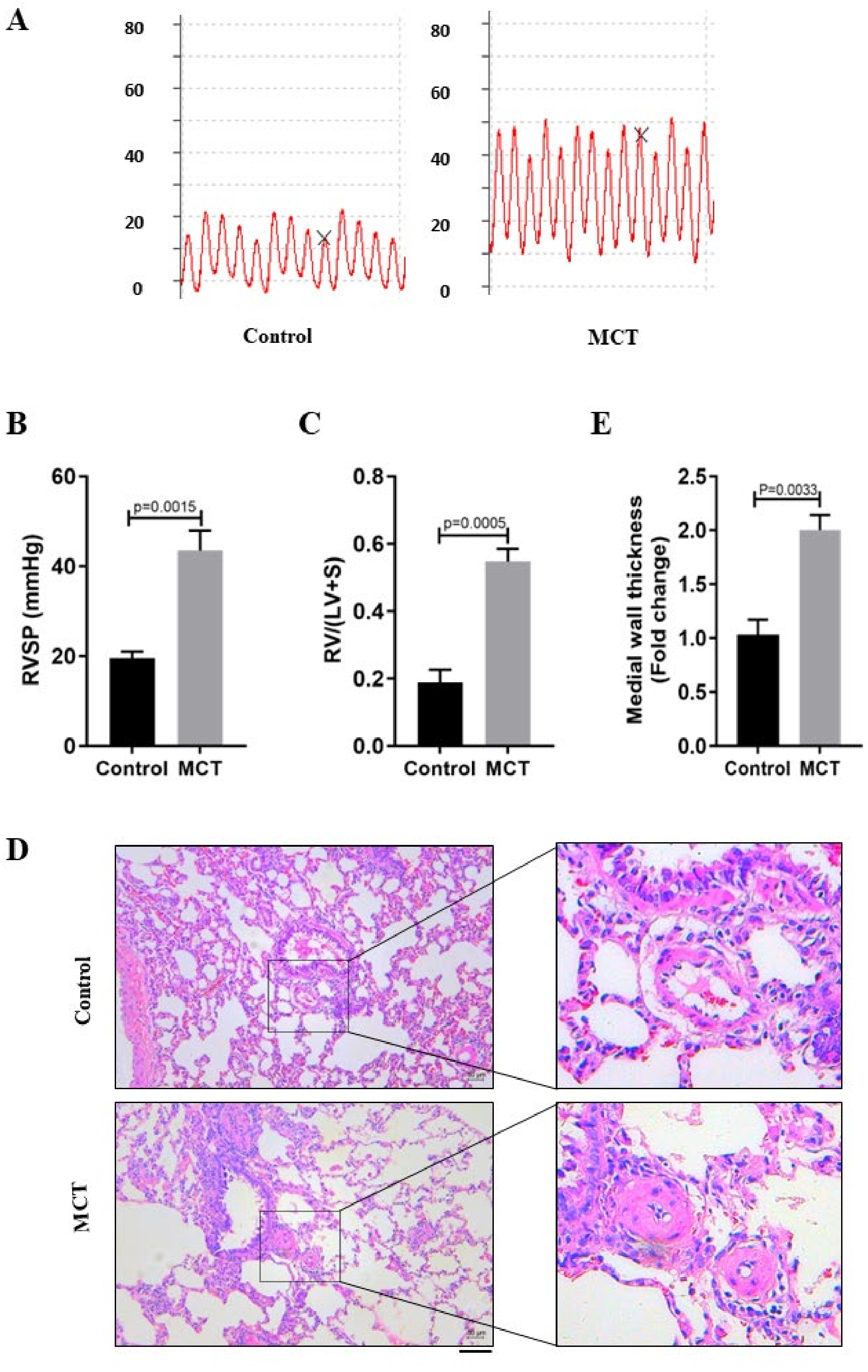

Supplemental Figure S1. PAH rat models validation. **(A-C)** Hemodynamic analyses of the control and the MCT-treatment groups. n=6 each. RVSP, right ventricular systolic pressure; RV/LV+S, an index of RV hypertrophy (Fulton index). (D and E) Hematoxylin and eosin staining of pulmonary artery in lung tissues (n = 6 each). Bar = 100 μm (left panel). Enlarged pulmonary artery (right panel).

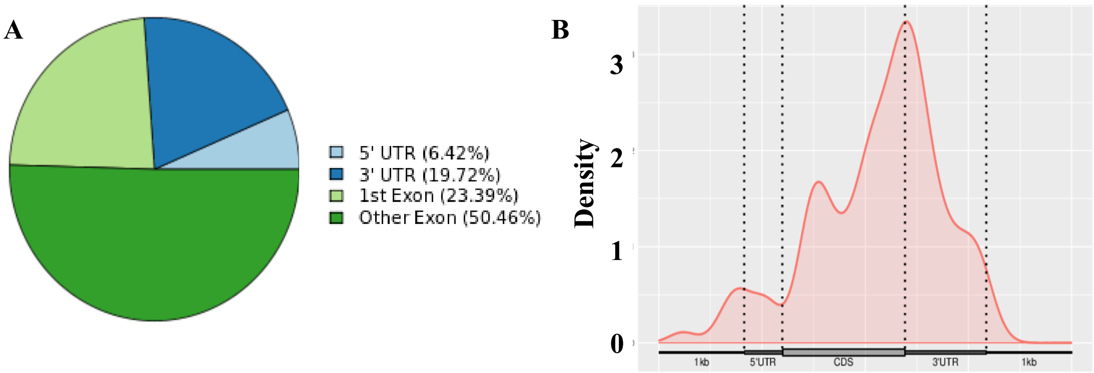

Supplemental Figure S2. Differential m^{6}A analysis in PAH. (A) Pie charts showing the percentage of m^{6}A peaks in four non-overlapping segments of transcripts. (B) Density curve showing the distribution of m^{6}A peaks across the transcripts. The transcript is divided into three parts, namely 5' UTR, CDS, and 3' UTR.

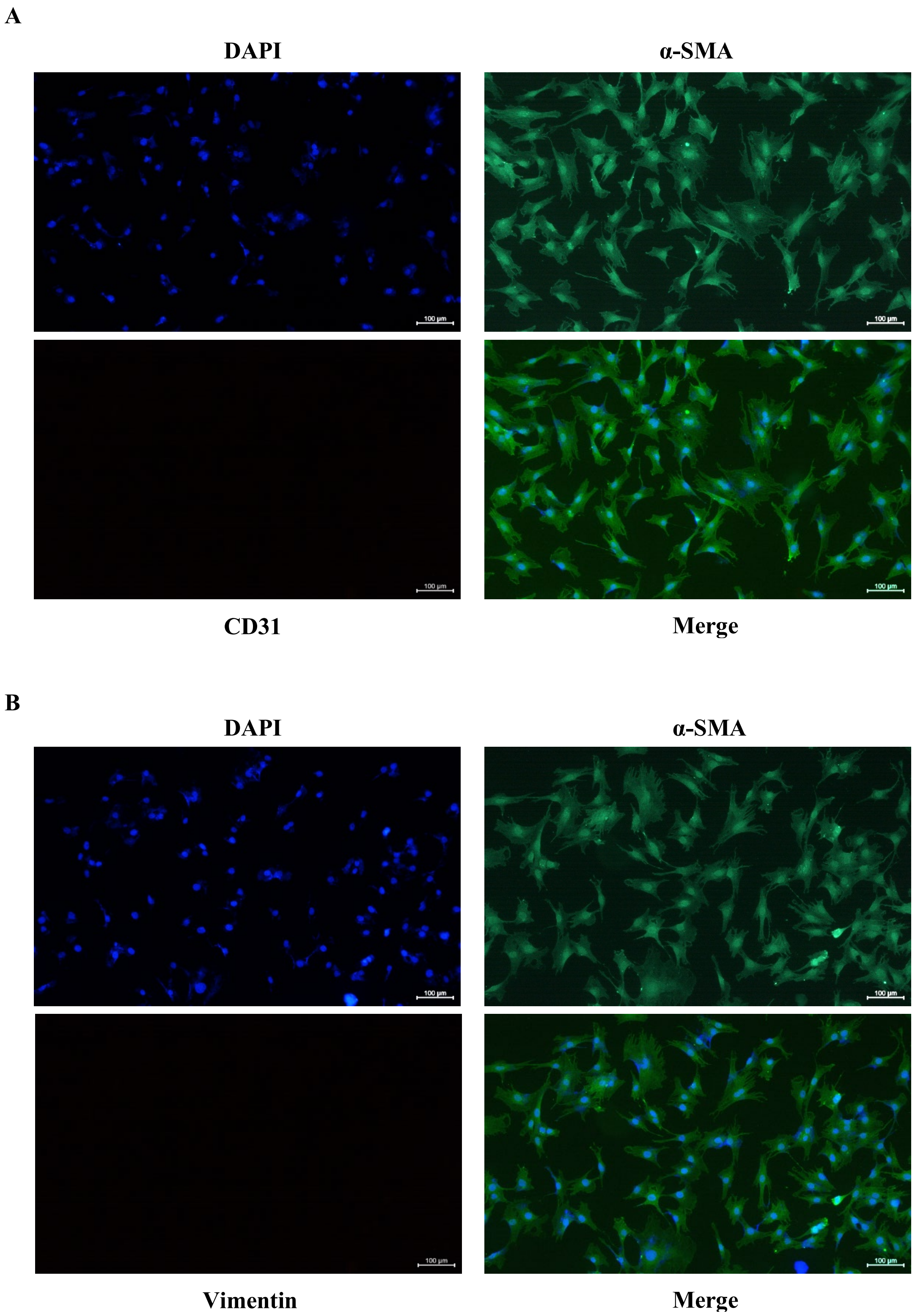

Supplemental Figure S3. PPI networks establishment and candidate genes identification. (A and B) Identify primary rat PASMCs. DAPI (blue), α-SMA (green), CD31 and Vimentin (red).

Supplemental Table S8. EdU cell numbers.

Biology repeat number	Cell group	Edu positive cell numbers	All cell numbers	Ratio (%)
1	siNC	20	155	12.9
	siLRPPRC	43	254	16.9
	PDGF+siNC	52	212	24.5
	PDGF+ siLRPPRC	56	187	30.0
2	siNC	18	134	13.4
	siLRPPRC	39	242	16.1
	PDGF+siNC	59	267	22.1
	PDGF+ siLRPPRC	67	215	31.2
3	siNC	12	115	10.4
	siLRPPRC	41	240	17.1
	PDGF+siNC	67	290	23.1
	PDGF+ siLRPPRC	76	251	30.3
